# When I say inclusion

**DOI:** 10.12688/mep.20155.1

**Published:** 2024-03-01

**Authors:** Rashmi Kusurkar

**Affiliations:** 1 Research in Education, Amsterdam UMC location Vrije Universiteit Amsterdam, De Boelelaan 1118, North Holland, 1081 HZ, The Netherlands; 2 LEARN! Research Institute for Learning and Education, Faculty of Psychology and Education, VU University Amsterdam, Amsterdam, The Netherlands; 3 Quality of Care, Amsterdam Public Health, Amsterdam, The Netherlands

**Keywords:** Inclusion; Health Professions Education; Medical Education

## Abstract

There is no unified understanding of the concept of inclusion in the literature. Since inclusion is a hot topic in the current debates on equity diversity and inclusion, it is important to move towards creating a common understanding of this term. In this article I explore the concept of inclusion based on the current literature. When I say inclusion, it is not just a sense of belonging, but also the opportunity to participate and contribute meaningfully.

## Background

While preparing for my professor’s inaugural lecture ^1^ entitled, ‘The ABC of inclusion and motivation’, I searched extensively how inclusion has been defined, described or operationalized in the literature. ( Kusurkar, 2023) I realized that inclusion means different things to different people, regardless of them working in the same field or in different fields. There is no unified understanding of the term ‘inclusion’. Two separate articles, namely, “When I say…diversity’ and ‘When I say…diversity, equity and inclusion’ have been published in Health Professions Education, but still the concept of inclusion has not been dealt with in its entirety. ( Chiavaroli *et al.*, 2020; Rossi *et al.*, 2022) Considering that inclusion is currently a hot topic not only in Health Professions Education (HPE), but also in global health, it is important to create a common understanding of this concept. This will help us conduct research, organize education and design policies with regards to equity diversity inclusion (EDI) on a common ground.

### What is inclusion?

To cover the nuanced understanding of the concept, when I say inclusion it means:

*‘being included in a group which creates a sense of belonging as well as empowers individuals to contribute in an authentic and meaningful manner.’* ( Kusurkar, 2024)

### What are the literature-based arguments for arriving at this meaning of inclusion?

Inclusion has been included in the literature on psychological safety. Clark describes four stages of psychological safety: inclusion safety, learner safety, contributor safety and challenger safety. Inclusion safety is the first stage and the first step towards innovation. ( Clark, 2020) Clark equates inclusion to a sense of belonging and describes contributor safety as a further stage in which a person feels safe to contribute meaningfully. Challenger safety means that an individual feels safe enough to challenge a status quo, and this leads to innovation. Thus, Clark distinguishes contributing meaningfully from the concept of inclusion safety. ( Clark, 2020) So do Anjorin and Busari, while proposing a model in which advocacy and inclusion lead to a sense of belonging, improved academic performance and positive well-being.( Anjorin & Busari, 2023) While encouraging everyone to handle diversity, equity and inclusion as an interrelated system, Rossi *et al.*, also look at contributing meaningfully as an outcome of inclusion, rather than as an integral part of inclusion. ( Rossi *et al.*, 2022) Slootman *et al.*, define inclusion as, “a state in which all individuals, regardless of their identities, backgrounds or needs, can actively participate, and belong, in a setting.” This definition of inclusion does not fully overlap that of a sense of belonging, but includes participation as well. ( Slootman *et al.*, 2023)

**Figure 1.**
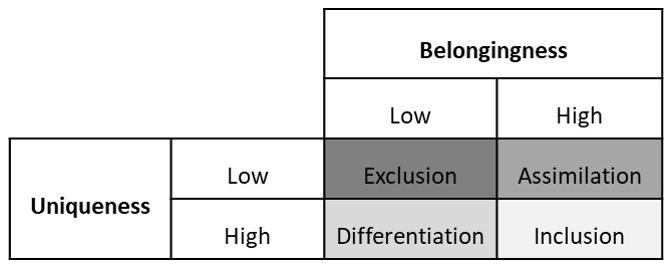
Shore *et al.*’s Inclusion Framework (Adapted from Shore *et al.*, 2011). *This figure depicts the 2X2 table based on Shore et al.*’ *s combinations of high or low belongingness on the one hand with high or low uniqueness on the other hand. Low belongingness ⴕ low uniqueness gives rise to exclusion. Low belongingness ⴕ high uniqueness leads to differentiation. High belongingness ⴕ low uniqueness leads to assimilation. High belongingness ⴕ high uniqueness leads to inclusion.*

For my EDI work, I start with the following concept: “Inclusion is the action or state of including or of being included within a group or structure. More than simply diversity and numerical representation, inclusion involves authentic and empowered participation and a true sense of belonging.” ( Annie. E. Casey Foundation) Inclusion refers to being included in a group which creates a sense of belonging as well as empowers individuals to contribute in an authentic and meaningful manner. ( Kusurkar, 2024) Thus, inclusion has two components: a sense of belonging and the empowerment to contribute meaningfully. ( Kusurkar, 2024) To understand why it is important that inclusion has these two components, we need to consider the inclusion framework proposed by Shore *et al.*, for work groups. ( Shore *et al.*, 2011) In this framework, the authors make a 2 X 2 table of belongingness and uniqueness. (See Figure 1) Belongingness means an individual feels that they are treated like an insider at work. Uniqueness means that an individual feels valued for their unique qualities at work.

- Low belongingness combined with low uniqueness means that an individual is neither treated like an insider nor valued for their unique qualities. This is labelled as *exclusion*.
- Low belongingness combined with high uniqueness means that an individual is not treated like an insider but is valued for their unique qualities. This is labelled as *differentiation*.
- High belongingness combined with low uniqueness means that an individual is treated like an insider but they are not valued for their unique qualities. They are expected to conform to the majority norms and values. This is labelled as *assimilation*.
- High belongingness combined with high uniqueness means that an individual is treated like an insider and valued for their unique qualities. This is labelled as inclusion.

Shore *et al.*, suggest that for inclusion, an individual should feel a sense of belonging while being valued to for their unique characteristics.

### The two or more players in inclusion

In my discussions with other scholars, often the following thought came up: “But I thought inclusion is an action that someone does to include the other. It is not the onus of the person who feels excluded to feel included. It purely depends on the person who acts to include the other”. I believe that we need to look at inclusion from two sides because the person who feels included or excluded also has agency. Inclusion involves at least two people (let’s say Tim and Sara), often more. Tim takes action to include Sara, and Sara is the one who needs to feel included. There could be a discordance between the two. Tim may feel that he has done everything to include Sara, but Sara may not feel included. Thus, inclusion is not merely the act of a person (Tim) who should be including the other (Sara). The person on the receiving end (in this case, Sara) is also intricately involved in this phenomenon of inclusion. She also has agency and can respond to inclusion or exclusion. This agency may be of differing degrees depending on the systemic barriers in the particular context, but nevertheless there is a possibility for agency, which cannot be ignored. Why and when is this important to take into consideration? This becomes especially important when EDI policies are created. The person on the receiving end of EDI opportunities may want them to be designed in a different manner than the person designing these policies. Thus including the people at the receiving end in designing the policy intended for them is important. It is in line with the principle: “For them, WITH them, about them” or “For them, *NOT* without them, about them”.

## Conclusion

Thus, when I say inclusion, it includes not just a sense of belonging, but also the opportunity to participate and contribute meaningfully. ( Kusurkar, 2023) Inclusion involves at least two players: the one who is inclusive and the other who is feeling or not feeling included. EDI policies should always be designed with the empowered participation of the people for whom they are being designed.

## References

[ref-1] Anjorin O Busari JO : Unpacking the social constructs of discrimination, othering, and belonging in Medical Schools. *Teach Learn Med.* Ahead of Print,2023:1–9. 10.1080/10401334.2023.2230211 37424255

[ref-2] Chiavaroli N Blitz J Cleland J : When I say …. Diversity. *Med Educ.* 2020;54(10):876–877. 10.1111/medu.14299 32725636

[ref-3] Clark TR : The 4 stages of psychological safety: Defining the path to inclusion and innovation.©Berrett-Koehler Publishers Inc., Oakland, CA,2020;1–18. Reference Source

[ref-4] Kusurkar RA : The ABC of inclusion and motivation.Inaugural lecture, Vrije Universiteit Amsterdam,2023; [Accessed 15 Oct 2023]. Reference Source

[ref-5] Kusurkar RA : Inclusive leadership in the health professions and health professions education. *BMJ Lead.* 2024: leader-2023-000868. 10.1136/leader-2023-000868 38182413 PMC12038138

[ref-6] Rossi AL Wyatt TR Huggett KN : When I say … diversity, equity and inclusion (DEI). *Med Educ.* 2022;56(7):701–702. 10.1111/medu.14812 35451160

[ref-7] Shore LM Randel AE Chung BG : Inclusion and Diversity in Work Groups: A Review and Model for Future Research. *J Manag.* 2011;37(4):1262–1289. 10.1177/0149206310385943

[ref-8] Slootman M Altes TK Domagała-Zyśk E : How to understand e-inclusion: the I-TPACK model.In: *A Handbook of e-Inclusion: Building Capacity for Inclusive Higher Education in Digital Environments.*Published by Knowledge Innovation Centre.2023;26–39. Reference Source

