# Supplementary material for: When I say inclusion in health professions education
Source: MedEdPublish (2016). 2024 Aug 20;14:7. Originally published 2024 Mar 1. [Version 3] doi: 10.12688/mep.20155.3 (PMC11170063; doi:10.12688/mep.20155.3)
Supplement: Peer review history

# Reviewer report: Reviewer response for version 3

**DOI:** 10.21956/mep.22011.r38462

**Published:** 2024-09-18

**Authors:** Maria Concetta Carruba

**Affiliations:** 1 Università Pegaso, Milan, Italy

Based on the author's revisions and comments on the previously provided suggestions, I have no further comments to add.

Is the topic of the opinion article discussed accurately in the context of the current literature?

Partly

Are arguments sufficiently supported by evidence from the published literature?

Partly

Are all factual statements correct and adequately supported by citations?

Partly

Are the conclusions drawn balanced and justified on the basis of the presented arguments?

Partly

Reviewer Expertise:

Inclusive Education, Technology for inclusion, TLC, UDL, Educational Technology, Accessibility

I confirm that I have read this submission and believe that I have an appropriate level of expertise to confirm that it is of an acceptable scientific standard.

# Reviewer report: Reviewer response for version 3

**DOI:** 10.21956/mep.22011.r38466

**Published:** 2024-09-12

**Authors:** Natallia B Hanssen

**Affiliations:** 1 Nord University, Bodø, Norway

Dear Author,

 Thank you very much for the revised version of your article. I believe the structure has become much stronger and clearer. I particularly appreciate the paragraph that discusses the meaning of inclusion and your effort to explore this phenomenon in the context of HPE.

Is the topic of the opinion article discussed accurately in the context of the current literature?

Partly

Are arguments sufficiently supported by evidence from the published literature?

Partly

Are all factual statements correct and adequately supported by citations?

Yes

Are the conclusions drawn balanced and justified on the basis of the presented arguments?

Partly

Reviewer Expertise:

Inclusion, inclusive education, special needs education, Interprofessional and family- professional collaboration, ECEC.

I confirm that I have read this submission and believe that I have an appropriate level of expertise to confirm that it is of an acceptable scientific standard.

# Reviewer report: Reviewer response for version 2

**DOI:** 10.21956/mep.21904.r37339

**Published:** 2024-06-12

**Authors:** Maria Concetta Carruba

**Affiliations:** 1 Università Pegaso, Milan, Italy

Thank you for letting me review this opinion article. I agree with the authors that inclusion is a widely debated and expansive phenomenon. In 1990, during the World Conference on Education for All in Jomtien, Thailand (March 5-9), it was emphasized that numerous students with Special Educational Needs (SEN) and marginalized students were excluded from education systems worldwide. It was declared that "everyone has a right to education" (UNESCO, 1990, p. 4). The Salamanca Statement (UNESCO, 1994) later defined inclusion as "the most effective means of combating discriminatory attitudes, creating welcoming communities, and building an inclusive society" (p. ix). Specific research fields provide extensive literature on this subject, particularly in education and within the Italian context, which boasts over 50 years of school inclusion. Inclusion values human diversity and emphasizes social justice and social equity. In this context, inclusion can be defined as a way of life, encompassing a set of core attitudes, beliefs, and values. Inclusion represents the method by which we manage our lives in social contexts and engage with diversity. Furthermore, the concept can be applied across various contexts, such as education and training, society, and the workplace, in different ways, leading to significant human impacts. It is crucial to remember that, according to the International Classification of Functioning, Disability and Health (ICF) by WHO, context, environment, and social interactions play a significant role in fostering inclusion or exclusion. Therefore, I recommend revising the work according to the World Health Organization's guidelines concerning the responsibility of the setting and the barriers that may be physical, architectural, cultural, or human. A society can only be deemed civilized if it cares for its most vulnerable members (Carruba, 2023) and strives for social equity across all aspects of life. This approach would enhance the completeness of the work and offer a more balanced perspective on how inclusion and exclusion impact the lives of everyone.

Is the topic of the opinion article discussed accurately in the context of the current literature?

Partly

Are arguments sufficiently supported by evidence from the published literature?

Partly

Are all factual statements correct and adequately supported by citations?

Partly

Are the conclusions drawn balanced and justified on the basis of the presented arguments?

Partly

Reviewer Expertise:

Inclusive Education, Technology for inclusion, TLC, UDL, Educational Technology, Accessibility

I confirm that I have read this submission and believe that I have an appropriate level of expertise to confirm that it is of an acceptable scientific standard, however I have significant reservations, as outlined above.

# Response

**Published:** 2024-07-23

**Authors:** Rashmi Kusurkar

**Affiliations:** Research in Education, Amsterdam UMC location Vrije Universiteit Amsterdam, Amsterdam, The Netherlands

I would like to thank the reviewer for their comments. I agree that inclusion is a topic that needs to be discussed beyond education and training. I think that by not emphasizing that this article is meant to discuss inclusion in the context of health professions education, has lead to confusion for the readers. To carry out revisions in line with the reviewer’s comments, I would like to revise the title of the article to “When I say inclusion in health professions education”. Right now this is implicit because I have chosen to publish in a health professions education journal. It would be better to make it explicit in the title.

**Reviewer's comment:**

 The reviewer says that the definition of inclusion in the Salamanca statement is “the most effective means of combating discriminatory attitudes, creating welcoming communities and building an inclusive society".

**Response:**

 The actual text in the Salamanca statement is, “ *regular schools with this inclusive orientation are the most effective means of combating discriminatory attitudes, creating welcoming communities, building an inclusive society and achieving education for all; moreover, they provide an effective education to the majority of children and improve the efficiency and ultimately the cost-effectiveness of the entire education system*”. This statement does not define inclusion and puts forth a limited idea of inclusion because it is limited to defining inclusion only for individuals with special education needs as seen in the opening statement, “ *More than 300 participants representing 92 governments and 25 international organizations met in Salamanca, Spain, from 7 to IO June 1994 to further the objective of Education for All by considering the fundamental policy shifts required to promote the approach of inclusive education, namely enabling schools to serve all children, particularly those with special educational needs*”.

 The title of the present article has been revised to, “ *When I say inclusion in health professions education*”.

# Reviewer report: Reviewer response for version 2

**DOI:** 10.21956/mep.21904.r37335

**Published:** 2024-06-11

**Authors:** Natallia B Hanssen

**Affiliations:** 1 Nord University, Bodø, Norway

Thank you very much for giving me an opportunity to review this opinion article.  I agree with the authors that inclusion is not a new concept, and it is  quite influential and widely debated phenomenon. To start, inclusion began to take off in 1990 at the World Conference on Education for All in Jomtien, Thailand (5–9 March). Delegates from 155 countries, as well as representatives from 150 governmental and nongovernmental organizations, acknowledged the exclusion of large numbers of vulnerable and marginalized students from education systems worldwide and asserted that ‘everyone has a right to education’ (UNESCO, 1990, p. 4).As seen, the Jomtien meeting moved inclusion  into the realm of diversity, giving the concept a broader vision because it ‘now incorporated a more extensive spectrum of concerns and discourses’ (Thomas, 2013, p. 474). In broad terms, the Salamanca Statement (UNESCO, 1994) outlined that inclusion is ‘the most effective means of combating discriminatory attitudes, creating welcoming communities and building an inclusive society" (p. ix).  For me,  inclusion based upon a humanistic ethos that values human diversity and emphasizes social justice and equity in society. In this sense, inclusion as ethos can be defined as a way of life, referring to a core set of attitudes, beliefs and values that gives credibility, coherence, vitality and identity to a community. Inclusion is the way we try to manage our collective lives and engage with diversity. As such, derived from this conceptualization of inclusion, we understand the concept of inclusive education as the challenges and possibilities to arouse a wanting to exist in the world of diversity

 and equality (Hanssen & Khitruk, 2021).Inclusion involves a subjective component, where the distinction between inclusion and exclusion may be blurred to some extent, and they have dynamic and mutual relationships that exist bundled together. For me, then, inclusion and exclusion can appear as a dilemma—a choice not between right and wrong, but between right and right. 

 To illustrate this,  the understanding  inclusion  can be likened to making a sculpture – if you only listen to your idea and not the stone, it will break. If you push too hard to overcome resistance, the stone will crumble.

 Based on my reflection, it would be fruitful to add a more nuanced discussion about how to understand inclusion from the perspective of medical and health professions education (HPE), providing specific examples within the HPE context.

 Best regards, Natallia B Hanssen, Nord University 

**Referenses:**

 Hanssen, N.B & Khitruk, V ( 2021).   Understanding Inclusion and Inclusive Education for  

  Students with SEN—Ideals and Reality In Natallia Bahdanovich Hanssen, Sven-Erik   

  Hansèn & Kristin Ström ( Eds.) *Dialogues between Northern and Eastern Europe on*

*the Development of Inclusion : Theoretical and Practical Perspectives. * Routledge

 UNESCO. (1990). World declaration on education for all and framework for action to meet basic learning needs. Adopted at the World Conference on Education for All Meeting Basic Learning Needs, Jomtien, Thailand, 5–9 March 1990.

 UNESCO. (1994). The Salamanca statement and framework for action on special needs

 education. In World Conference on Special Needs Education: Access and Quality, Salamanca,

 Spain, 7–10 June 1994.

 Thomas, G. (2013). A review of thinking and research about inclusive education policy with

 suggestions of a new kind of inclusive thinking. British Educational Research Journal, 39,

 473–490.

 title=Construction navale à franc-bord en Méditerranée et Atlantique (XIVe–XVIIe siècle) et “signatures architecturales”: Une première approche archéologique, Méditerranée Antique. Pêche, Navigation, Commerce|authors=Rieth/E|source=Paris: CTHS|year=1998

Is the topic of the opinion article discussed accurately in the context of the current literature?

Partly

Are arguments sufficiently supported by evidence from the published literature?

Partly

Are all factual statements correct and adequately supported by citations?

Yes

Are the conclusions drawn balanced and justified on the basis of the presented arguments?

Partly

Reviewer Expertise:

Inclusion, inclusive education, special needs education, Interprofessional and family- professional collaboration, ECEC.

I confirm that I have read this submission and believe that I have an appropriate level of expertise to confirm that it is of an acceptable scientific standard, however I have significant reservations, as outlined above.

## References

[1] : Inclusion and Inclusive Education in Russia: Analysis of Legislative and Strategic Documents at the State Level between 2012–2014. *Education Sciences* .2024;14(3) : 10.3390/educsci14030312 10.3390/educsci14030312

[2] World Declaration on Education for All and Framework for Action to Meet Basic Learning Needs. *UNESCO* .1990;

[3] The Salamanca Statement and Framework for Action on Special Needs Education. *UNESCO* .1994;

[4] : A review of thinking and research about inclusive education policy, with suggestions for a new kind of inclusive thinking. *British Educational Research Journal* .2013;39(3) : 10.1080/01411926.2011.652070 473-490 10.1080/01411926.2011.652070

# Response

**Published:** 2024-07-23

**Authors:** Rashmi Kusurkar

**Affiliations:** Research in Education, Amsterdam UMC location Vrije Universiteit Amsterdam, Amsterdam, The Netherlands

I would like to thank the reviewer for their comments.

**Reviewer's comment:**

 The reviewer says that the definition of inclusion in the Salamanca statement is “the most effective means of combating discriminatory attitudes, creating welcoming communities and building an inclusive society".

**Response:**

 The actual text in the Salamanca statement is, “ *regular schools with this inclusive orientation are the most effective means of combating discriminatory attitudes, creating welcoming communities, building an inclusive society and achieving education for all; moreover, they provide an effective education to the majority of children and improve the efficiency and ultimately the cost-effectiveness of the entire education system*”. This statement does not define inclusion and puts forth a limited idea of inclusion because it is limited to defining inclusion only for individuals with special education needs as seen in the opening statement, “ *More than 300 participants representing 92 governments and 25 international organizations met in Salamanca, Spain, from 7 to IO June 1994 to further the objective of Education for All by considering the fundamental policy shifts required to promote the approach of inclusive education, namely enabling schools to serve all children, particularly those with special educational needs*”. I have added specific examples of the importance of inclusion in HPE along with references.

# Reviewer report: Reviewer response for version 2

**DOI:** 10.21956/mep.21904.r36980

**Published:** 2024-06-11

**Authors:** Paolo C Martin

**Affiliations:** 1 Uniformed Services University, Bethesda,, Maryland, USA

Thank you for the opportunity to review this article, which aims to bring clarity to the term “inclusion.” Topics in the field of equity, diversity, and inclusion (EDI) are important to understand particularly in health professions education where health professions learners from minoritized backgrounds as well as the care and health outcomes of minoritized patient communities are affected by EDI policies and practices. With respect to those issues, the premise of this article—understanding the idea of “inclusion”—is an important one to consider.

 I appreciate the author’s effort to draw attention to some current understandings of the term “inclusion,” especially with respect to other related constructs such as psychological safety (Clark, 2020), and motivation (Ryan and Deci, 2000). Indeed, the fostering inclusive environments is important to developing teaching and learning environments that are psychological safe and that build intrinsic motivation. I also appreciate the author’s focus on how a person’s unique identities must be valued and engaged through empowered participation as couched in Shore’s (2011) Inclusion Framework. I also agree with the author that inclusion should be considered with respect to the interactional space between the person at risk of being excluded and the person or persons with more privileged and normalized identities. These are all important considerations, and I am glad that the author had edited the original version of the article to relate these considerations to HPE issues such as the disparities in HPE research, especially with respect to the disenfranchisement of research from the Global South in favor of research from the Global North.

 Despite attention to those important considerations, I believe this article should not be approve in its current form for the following reasons:

 1. The article does not seem to strongly add to the “nuanced understanding of the concept” of inclusion, which the author has written as the aim of the article. The facets of belonging with careful consideration for engaged participation, which aims to set apart this article’s definition of inclusion is a point that others seem to have made when considering inclusion. Indeed, the author cites references that incorporates those points. Others works have suggested that the history of the term “inclusion” from the field of special education needs (SEN) to the Civil Rights Movement in the United States has emphasized those aspects of inclusion (Hodkinson, 2011). With respect to Psychological Safety in HPE, McClintock et al (2022) have also noted the importance of participation and belonging in clinical learning environments.

 2. It is unclear in the opening section about how the professor’s lecture and the author's interest to know how inclusion has been defined exemplifies *why* understanding the topic of “inclusion” is important to understand. For example, why is it important to tease it out from equity, diversity, and inclusion? If it is a hot topic, why are the discourses surrounding this topic not enough? One way to improve the article might be to apply a critical stance at how it has been applied in HPE and why those ways of applying definitions of “inclusion” have been problematic for patients and for HPE learners, educators, researchers. The issue can be followed by an argument for how the authors’ proposed definition improves current definitions in a way to better serve those impacted by less-than-ideal definitions of “inclusion.” The article could also relate the importance of inclusion with respect to fighting systemic biases that work to exclude marginalized identities. Indeed, it would help to cover how definitions of inclusion could still be influenced by those deeply rooted biases that protect privileged classes through performative allyship (Kutlaca and Rodke, 2023) to help understand how the author’s definition of “inclusion” works against performative allyship?

 3. While the article mentions the importance of inclusion to HPE topics such as disparities in research, it still fails to connect the literature on inclusion with how it has played out in the field of HPE as part of the discussion of the current literature as well as the importance of inclusion to other HPE topics other than disparities in HPE research. Indeed, as it stands the current version of this article does not seem to add much to the works that the author has previously published related to this topic and has cited in this article.

Is the topic of the opinion article discussed accurately in the context of the current literature?

Partly

Are arguments sufficiently supported by evidence from the published literature?

Partly

Are all factual statements correct and adequately supported by citations?

Yes

Are the conclusions drawn balanced and justified on the basis of the presented arguments?

Partly

Reviewer Expertise:

Whiteness Theory, DEI (Diversity, Equity, Inclusion), Marginalized Voices, Critical Research Methods, Critical Childhood Studies, Child Rights to Provisions, Protection, and Participation.

I confirm that I have read this submission and believe that I have an appropriate level of expertise to state that I do not consider it to be of an acceptable scientific standard, for reasons outlined above.

## References

[1] : The 4 stages of psychological safety: defining the path to inclusion and innovation. *Berrett-Koehler* .2020;

[2] : Inclusion and diversity in work groups: a review and model for future research. *J Manag* .2011;

[3] : Self-determination theory and the facilitation of intrinsic motivation, social development, and well-being. *Am Psychol* .2000;55(1) : 10.1037//0003-066x.55.1.68 68-78 10.1037//0003-066x.55.1.68 11392867

[4] : Inclusion: A Defining Definition?. *Power and Education* .2011;3(2) : 10.2304/power.2011.3.2.179 179-185 10.2304/power.2011.3.2.179

[5] : Clinician Teacher as Leader: Creating Psychological Safety in the Clinical Learning Environment for Medical Students. *Acad Med* .2022;97(11S) : 10.1097/ACM.0000000000004913 S46-S53 10.1097/ACM.0000000000004913 35947474

[6] : Towards an understanding of performative allyship: Definition, antecedents and consequences. *Social and Personality Psychology Compass* .2023;17(2) : 10.1111/spc3.12724 10.1111/spc3.12724

# Response

**Published:** 2024-07-23

**Authors:** Rashmi Kusurkar

**Affiliations:** Research in Education, Amsterdam UMC location Vrije Universiteit Amsterdam, Amsterdam, The Netherlands

Thank you for the opportunity to review this article, which aims to bring clarity to the term “inclusion.” Topics in the field of equity, diversity, and inclusion (EDI) are important to understand particularly in health professions education where health professions learners from minoritized backgrounds as well as the care and health outcomes of minoritized patient communities are affected by EDI policies and practices. With respect to those issues, the premise of this article—understanding the idea of “inclusion”—is an important one to consider.

 I appreciate the author’s effort to draw attention to some current understandings of the term “inclusion,” especially with respect to other related constructs such as psychological safety (Clark, 2020), and motivation (Ryan and Deci, 2000). Indeed, the fostering inclusive environments is important to developing teaching and learning environments that are psychological safe and that build intrinsic motivation. I also appreciate the author’s focus on how a person’s unique identities must be valued and engaged through empowered participation as couched in Shore’s (2011) Inclusion Framework. I also agree with the author that inclusion should be considered with respect to the interactional space between the person at risk of being excluded and the person or persons with more privileged and normalized identities. These are all important considerations, and I am glad that the author had edited the original version of the article to relate these considerations to HPE issues such as the disparities in HPE research, especially with respect to the disenfranchisement of research from the Global South in favor of research from the Global North.

**Author response: **I would like to thank the reviewer for the appreciation of the paper.

**Reviewer’s comment:** Despite attention to those important considerations, I believe this article should not be approve in its current form for the following reasons:

1. The article does not seem to strongly add to the “nuanced understanding of the concept” of inclusion, which the author has written as the aim of the article. The facets of belonging with careful consideration for engaged participation, which aims to set apart this article’s definition of inclusion is a point that others seem to have made when considering inclusion. Indeed, the author cites references that incorporates those points. Others works have suggested that the history of the term “inclusion” from the field of special education needs (SEN) to the Civil Rights Movement in the United States has emphasized those aspects of inclusion (Hodkinson, 2011). With respect to Psychological Safety in HPE, McClintock et al (2022) have also noted the importance of participation and belonging in clinical learning environments.
**Response: **Nembhard & Edmondson (2006) have proposed the following definition of leader inclusiveness, “Words and deeds by a leader or leaders that indicate an invitation and appreciation for others’ contributions”. Nembhard & Edmondson’s article is about power hierarchies between healthcare workers and the psychological safety in that context. McClintock et al (2022) use Edmondson’s concept of psychological safety, focusing on active and safe participation, but does not include sense of belonging. The Salamanca statement talks about inclusive education with a specific focus on special education needs, but does not define inclusion as such. Hodkinson (2011) also focuses his entire article on inclusion in relation to special education needs. In the present article, I try to present the joint concept of sense of belonging and meaningful participation as inclusion, which is an addition to the literature. *Nembhard IA, Edmundson AC. Making it safe: The effects of leader inclusiveness and professional status on psychological safety and improvement efforts in health care teams. J Organiz Behav 2006;27:941–966.* *McClintock AH, Fainstad TL, Jauregui J. Clinician Teacher as Leader: Creating Psychological Safety in the Clinical Learning Environment for Medical Students. Acad Med 2022;97(11S):S46-S53.* *UNESCO. (1994). The Salamanca statement and framework for action on special needs* *education. In World Conference on Special Needs Education: Access and Quality, Salamanca,* *Spain, 7–10 June 1994.* *Hodkinson A. Inclusion: a defining definition? Pow Educ 2011;3(2):179-185.*
2. **Reviewer’s comment: **It is unclear in the opening section about how the professor’s lecture and the author's interest to know how inclusion has been defined exemplifies *why* understanding the topic of “inclusion” is important to understand. For example, why is it important to tease it out from equity, diversity, and inclusion? If it is a hot topic, why are the discourses surrounding this topic not enough? One way to improve the article might be to apply a critical stance at how it has been applied in HPE and why those ways of applying definitions of “inclusion” have been problematic for patients and for HPE learners, educators, researchers. The issue can be followed by an argument for how the authors’ proposed definition improves current definitions in a way to better serve those impacted by less-than-ideal definitions of “inclusion.” The article could also relate the importance of inclusion with respect to fighting systemic biases that work to exclude marginalized identities. Indeed, it would help to cover how definitions of inclusion could still be influenced by those deeply rooted biases that protect privileged classes through performative allyship (Kutlaca and Rodke, 2023) to help understand how the author’s definition of “inclusion” works against performative allyship?
**Response: **I agree with the reviewer that more explanation on why this definition of inclusion is necessary would improve the article. I have therefore added the following paragraph in the article: ***Why is it important to clarify this concept in HPE?*** *Clarifying the definition of inclusion in HPE is important in order to find ways of measuring inclusion quantitatively as well as studying it qualitatively. The possibility to measure the extent of inclusion will allow for testing interventions and measuring their effects through pre and post intervention scores. A description of inclusion that is based on systemic barriers does not allow for such measurements. Considering a definition based on the psychological aspect of inclusion is therefore important in HPE. *
  I have also added a paragraph on critical reflections on the choices made in the article: ***A critical reflection on the choices made in this article*** *This article focuses on inclusion rather than exclusion. The reason for this choice is that many people who may be in a privileged position want to facilitate and practice inclusion. They  would like to take-away tips on facilitating inclusion, but with the lack of consensus on what inclusion means, they don’t have a working concept to begin with.* *This article also focuses on the psychological aspect of inclusion rather than the societal systemic barrier perspective. The reason for this choice is that there is a lot of literature on how to change systems, while there is relatively less availability of literature on how people can practice inclusion in their daily lives. This is not to say that studying exclusion and systemic barriers is not important; I myself have published extensively on this topic. It is just not the focus in this article. *  
3. **Reviewer’s comment: **While the article mentions the importance of inclusion to HPE topics such as disparities in research, it still fails to connect the literature on inclusion with how it has played out in the field of HPE as part of the discussion of the current literature as well as the importance of inclusion to other HPE topics other than disparities in HPE research. Indeed, as it stands the current version of this article does not seem to add much to the works that the author has previously published related to this topic and has cited in this article.
**Response: **With due respect to the reviewer I disagree. The whole idea of writing this article was to propose inclusion as a combination of sense of belonging and the empowerment for meaningful participation, which has not been done before.

# Reviewer report: Reviewer response for version 2

**DOI:** 10.21956/mep.21904.r37237

**Published:** 2024-06-07

**Authors:** Diantha Soemantri

**Affiliations:** 1 Universitas Indonesia, Jakarta, Indonesia

I would like to thank you the author for the revision of the paper. I believe the revisions that the author has provided are sufficient. In this current form, the manuscript is comprehensive, well supported with references, and the arguments in the article are succintly justified. The manuscript can provide us with the latest concepts of inclusion and its implications toward medical education.

Is the topic of the opinion article discussed accurately in the context of the current literature?

Yes

Are arguments sufficiently supported by evidence from the published literature?

Yes

Are all factual statements correct and adequately supported by citations?

Yes

Are the conclusions drawn balanced and justified on the basis of the presented arguments?

Yes

Reviewer Expertise:

NA

I confirm that I have read this submission and believe that I have an appropriate level of expertise to confirm that it is of an acceptable scientific standard.

# Reviewer report: Reviewer response for version 2

**DOI:** 10.21956/mep.21904.r37337

**Published:** 2024-06-07

**Authors:** Athanasios Koutsoklenis

**Affiliations:** 1 Democritus University of Thrace, Alexandroupolis, Greece

**Review of “When I say inclusion” by Rashmi Kusurkar**

 Dear Editor and Author,

 Thank you for the opportunity to review this paper. I respectfully submit my comments below.

 Comment #1. The author has adapted a definition of inclusion that heavily emphasizes two psychological aspects: a sense of belonging and empowerment, which concern the individual. However, inclusion is not only about these aspects; it is primarily about removing structural barriers to participation and supporting equity at all levels. The heavy emphasis on the psychological aspect of inclusion is also evident in a later part of the paper, which refers to the work of Clark (2020). Adopting a broader view on inclusion we have recently provided the following definition: “Inclusion in education is a globally shared principle, and its aim is to guarantee, secure, and promote the equality and equity of all people by removing barriers to learning and social participation. It is not an outcome but an ongoing principled process that requires commitment and dedication, and this is carried out through aligning various inclusive policies, cultural elements, and institutional, discursive, social, and pedagogical practices.” (Honkasilta and Koutsoklenis, 2024).

 Comment #2. In a paper titled “When I Say Inclusion”, the author cites her own very recent work for the definition of inclusion, despite not being an expert on the subject, whether in social or educational contexts.

 Comment #3. The authors states, “I realized that inclusion means different things to different people, regardless of them working in the same field or in different fields. There is no unified understanding of the term ‘inclusion’.” However, the author does not provide any explanation for why this happens.

 Comment #4. The explanation provided by the author for the benefits of a common conceptualization and understanding is superficial and lacks scientific and political justification. The author states, “Considering that inclusion is currently a hot topic not only in Health Professions Education (HPE), but also in global health, it is important to create a common understanding of this concept”. Is it important because it is a ‘hot topic’ or because so many people are excluded, segregated, marginalized, stigmatized, and so on due to their characteristics or background?

 Comment #5. The author adopts a celebratory view on inclusion (e.g., "Uniqueness means that an individual feels valued for their unique qualities at work," "For them, WITH them, about them,” or “For them, NOT without them, about them.”). The paper lacks any effort to explain why people are excluded.

 Comment #6. In the example provided in the section titled ‘The two or more players in inclusion’ the author emphasizes the role of agency. Emphasizing the role of agency without even discussing structural barriers again means an individualized perspective on inclusion which means that for example, disabled, Black or indigenous people “need to feel included” because “There could be a discordance between the two”. Is exclusion simply a matter of feelings or discordance?

 Comment #7. The basic argument (i.e., “Thus, when I say inclusion, it includes not just a sense of belonging but also the opportunity to participate and contribute meaningfully”) is repeated several times throughout the paper. The argument is based on Shore et al.'s (2011) inclusion framework; no justification is provided for why this framework is better than others.

 Comment #8. This paper aims at suggesting a conceptualization of 'inclusion' in the context of medical and health professions education (HPE). In the section titled “Examples in the HPE context in which the definition of inclusion would be important” the author provides some general guidelines that can be applied to almost any educational framework and setting; they are not specific to any particular aspects of HPE.

 Sincerely,

 Athanasios Koutsoklenis

 Democritus University of Thrace

Is the topic of the opinion article discussed accurately in the context of the current literature?

Partly

Are arguments sufficiently supported by evidence from the published literature?

No

Are all factual statements correct and adequately supported by citations?

Partly

Are the conclusions drawn balanced and justified on the basis of the presented arguments?

No

Reviewer Expertise:

Inclusive education / Disability studies

I confirm that I have read this submission and believe that I have an appropriate level of expertise to state that I do not consider it to be of an acceptable scientific standard, for reasons outlined above.

## References

[1] : The 4 Stages of Psychological Safety. *Berrett-Koehler Publishers Inc ., Oakland, CA,* .2020;

[2] : A. International Perspectives on Inclusion in Education: Exploring Common Ground from Different Angles. *(Multidisciplinary Digital Publishing Institute* .2024;

[3] : Inclusion and Diversity in Work Groups: A Review and Model for Future Research. *Journal of Management* .2011;37(4) : 10.1177/0149206310385943 1262-1289 10.1177/0149206310385943

# Response

**Published:** 2024-07-23

**Authors:** Rashmi Kusurkar

**Affiliations:** Research in Education, Amsterdam UMC location Vrije Universiteit Amsterdam, Amsterdam, The Netherlands

1. **Reviewer's comment:**. The author has adapted a definition of inclusion that heavily emphasizes two psychological aspects: a sense of belonging and empowerment, which concern the individual. However, inclusion is not only about these aspects; it is primarily about removing structural barriers to participation and supporting equity at all levels. The heavy emphasis on the psychological aspect of inclusion is also evident in a later part of the paper, which refers to the work of Clark (2020). Adopting a broader view on inclusion we have recently provided the following definition: “Inclusion in education is a globally shared principle, and its aim is to guarantee, secure, and promote the equality and equity of all people by removing barriers to learning and social participation. It is not an outcome but an ongoing principled process that requires commitment and dedication, and this is carried out through aligning various inclusive policies, cultural elements, and institutional, discursive, social, and pedagogical practices.” (Honkasilta and Koutsoklenis, 2024).
**Response: **I agree with the reviewer that the concept of inclusion explained in this article is mainly about the psychological aspect of inclusion. I also agree that for true inclusion, structural barriers need to be removed and systems need to be disrupted. Because I would like to measure inclusion in HPE and set up interventions in HPE curricula, I decided to focus my concept of inclusion on its psychological aspect. I have added this information to the article:  
***What is inclusion?*** *To cover the nuanced understanding of the concept, when I say inclusion (from the psychological aspect) it means:* *‘being included in a group which creates a sense of belonging as well as empowers individuals to contribute in an authentic and meaningful manner.’ (* *Kusurkar, 2024* *) *   I have also added this to the abstract: *There is no unified understanding of the concept of inclusion in the literature. Because of the growing reports of exclusion and marginalization in HPE, and the reports of inequitable health provision in global health, inclusion is currently a widely discussed topic in Health Professions Education (HPE) as well as global health.  In this article I explore the concept of inclusion based on the current literature, mainly from the psychological aspect. When I say inclusion, it is not just a sense of belonging, but also the opportunity to participate and contribute meaningfully.*   I have also added it in the following text: *For my EDI work, I start with the following concept, which is focused on the psychological aspect of inclusion: “Inclusion is the action or state of including or of being included within a group or structure. More than simply diversity and numerical representation, inclusion involves authentic and empowered participation and a true sense of belonging.” (* *Annie. E. Casey Foundation* *) Inclusion refers to being included in a group which creates a sense of belonging as well as empowers individuals to contribute in an authentic and meaningful manner. (* *Kusurkar, 2024* *)* I have also added the following paragraph to the article:  
***Why is it important to clarify this concept in HPE?*** *Clarifying the definition of inclusion in HPE is important in order to find ways of measuring inclusion quantitatively as well as studying it qualitatively. The possibility to measure the extent of inclusion will allow for testing interventions and measuring their effects through pre and post intervention scores. A description of inclusion that is based on systemic barriers does not allow for such measurements. Considering a definition based on the psychological aspect of inclusion is therefore important in HPE.  *  
*I have also added it in the conclusion:* ***Conclusion*** *Thus, when I say inclusion, from the psychological aspect, it includes not just a sense of belonging, but also the opportunity to participate and contribute meaningfully. (* *Kusurkar, 2023* *) Inclusion involves at least two players: the one who is inclusive and the other who is feeling or not feeling included. EDI policies should always be designed with the empowered participation of the people for whom they are being designed.*    
2. **Reviewer's comment:** In a paper titled “When I Say Inclusion”, the author cites her own very recent work for the definition of inclusion, despite not being an expert on the subject, whether in social or educational contexts.
**Response: **I am surprised with the personal tone and judgement passed in this comment about me without having the correct background information. I should expect a formal apology from the reviewer for making such a statement. This type of feedback is neither scientific nor acceptable in the scientific world.
3. **Reviewer's comment:** The authors states, “I realized that inclusion means different things to different people, regardless of them working in the same field or in different fields. There is no unified understanding of the term ‘inclusion’.” However, the author does not provide any explanation for why this happens.
**Response: **The statement that the reviewer quotes here is explained in the following statements. To make it clearer I have revised this statement to: “I realized that inclusion means different things to different people, regardless of them working in the same field or in different fields. There is no unified understanding of the term ‘inclusion’ *in the literature*.”
4. **Reviewer's comment:** The explanation provided by the author for the benefits of a common conceptualization and understanding is superficial and lacks scientific and political justification. The author states, “Considering that inclusion is currently a hot topic not only in Health Professions Education (HPE), but also in global health, it is important to create a common understanding of this concept”. Is it important because it is a ‘hot topic’ or because so many people are excluded, segregated, marginalized, stigmatized, and so on due to their characteristics or background?
**Response: **I would like to thank the reviewer for this feedback. The sentence comes across in a different way than was originally intended. I merely meant to say that inclusion is currently being discussed extensively in HPE. The intention was never to ignore marginalized people. I have reworded the sentence as: “Because of the growing reports of exclusion and marginalization in HPE, and the reports of inequitable health provision in global health, inclusion is currently a widely discussed topic in Health Professions Education (HPE) as well as global health. It is, therefore, important to create a common understanding of this concept. This will help us conduct research, organize education and design policies with regards to equity diversity inclusion (EDI) on a common ground.” I have also revised this in the abstract.
5. **Reviewer's comment:** The author adopts a celebratory view on inclusion (e.g., "Uniqueness means that an individual feels valued for their unique qualities at work," "For them, WITH them, about them,” or “For them, NOT without them, about them.”). The paper lacks any effort to explain why people are excluded.
**Response: **I agree with the reviewer's comment. This paper is focused on inclusion rather than exclusion. I have added a critical reflection on the choices made in the paper before the conclusion.  
***A critical reflection on the choices made in this article*** *This article focuses on inclusion rather than exclusion. The reason for this choice is that many people who may be in a privileged position want to facilitate and practice inclusion. They  would like to take-away tips on facilitating inclusion, but with the lack of consensus on what inclusion means, they don’t have a working concept to begin with.* *This article also focuses on the psychological aspect of inclusion rather than the societal systemic barrier perspective. The reason for this choice is that there is a lot of literature on how to change systems, while there is relatively less availability of literature on how people can practice inclusion in their daily lives. This is not to say that studying exclusion and systemic barriers is not important; I myself have published extensively on this topic. It is just not the focus in this article.*
6. **Reviewer's comment:** In the example provided in the section titled ‘The two or more players in inclusion’ the author emphasizes the role of agency. Emphasizing the role of agency without even discussing structural barriers again means an individualized perspective on inclusion which means that for example, disabled, Black or indigenous people “need to feel included” because “There could be a discordance between the two”. Is exclusion simply a matter of feelings or discordance?
**Response: **I agree with the reviewer that by not discussing structural barriers and exploring the role of agency, this article may lead the readers to draw unintended and wrong conclusions. What I intended to say was that if a person thinks they are being inclusive, it does not necessarily mean that the person who should feel included actually feels included. The person who is trying to be inclusive can conclude that they are being inclusive only if then person at the receiving end experiences inclusion. I have revised the said paragraph to reflect my thinking and also added a paragraph on a critical reflection on the choices made in the present paper:
***The two or more players in inclusion*** *In my discussions with other scholars, often the following thought came up: “But I thought inclusion is an action that someone does to include the other. It is not the onus of the person who feels excluded to feel included. It purely depends on the person who acts to include the other”. I completely agree that it is not the onus of the person who feels excluded to feel included. But looking at inclusion only in this way might have an unintended consequence. While creating EDI policies, such a perspective can lead to marginalized groups being left out of the policy making process. The person on the receiving end of EDI opportunities (a marginalized group member) may want them to be designed in a different manner than the person (majority group member) designing these policies. Thus including the people at the receiving end in designing the policy intended for them is important. It is in line with the principle: “For them, WITH them, about them” or “For them, NOT without them, about them”.* *Thus, I believe that we need to look at inclusion from two sides because the person who feels included or excluded also has an opinion and agency. Inclusion involves at least two people (let’s say Tim and Sara), often more. Tim takes action to include Sara, and Sara is the one who needs to feel included. There could be a discordance between the two. Tim may feel that he has done everything to include Sara (even if he has not), but Sara may not feel included. It is the personal experience of Sara which is more important in this case. Thus, inclusion is not merely the act of a person (Tim) who should be including the other (Sara). The person on the receiving end (in this case, Sara) is also intricately involved in this phenomenon of inclusion. She also has an opinion and agency, and can respond to inclusion or exclusion or choose not to respond. From the mere absence of a response it cannot be inferred as the person feels included. Thus, finding out if the person on the receiving end feels included is key in the process of inclusion. *
***A critical reflection on the choices made in this article*** *This article focuses on inclusion rather than exclusion. The reason for this choice is that many people who may be in a privileged position want to facilitate and practice inclusion. They  would like to take-away tips on facilitating inclusion, but with the lack of consensus on what inclusion means, they don’t have a working concept to begin with.* *This article also focuses on the psychological aspect of inclusion rather than the societal systemic barrier perspective. The reason for this choice is that there is a lot of literature on how to change systems, while there is relatively less availability of literature on how people can practice inclusion in their daily lives. This is not to say that studying exclusion and systemic barriers is not important; I myself have published extensively on this topic. It is just not the focus in this article.*
7. **Reviewer's comment:** The basic argument (i.e., “Thus, when I say inclusion, it includes not just a sense of belonging but also the opportunity to participate and contribute meaningfully”) is repeated several times throughout the paper. The argument is based on Shore et al.'s (2011) inclusion framework; no justification is provided for why this framework is better than others.
**Response: **Based on reviewer Nathalie Hanssen’s comments, I have added that my depiction of inclusion is about the psychological aspect of inclusion. This is also the reason why my concept of inclusion is based on Shore et al.’s framework rather than any other framework use for inclusion. I have added the following explanation in the manuscript: *Shore et al.’s framework is based on the psychological aspect of inclusion and exclusion, and is, therefore, relevant for this article.*
8. **Reviewer's comment:** This paper aims at suggesting a conceptualization of 'inclusion' in the context of medical and health professions education (HPE). In the section titled “Examples in the HPE context in which the definition of inclusion would be important” the author provides some general guidelines that can be applied to almost any educational framework and setting; they are not specific to any particular aspects of HPE.
**Response: **I agree with the reviewer that the suggestions made can be applied to any educational framework. In order to make them more relevant to HPE, I have added references to HPE articles that have reported problems in the mentioned topics. In HPE the definition of inclusion would be important in the areas following areas:

- Admission to HPE, including selection for HPE, in order to ensure that all populations from the society are represented in the HPE student population. (Mulder et al. 2023)
- During HPE:
  - for creating the optimal learning environment in classrooms, small group education, practicals and in workplace learning contexts, in which all students can flourish, (Isik et al. 2021) and
  - for creating inclusive curricular content. (Muntinga et al. 2016)
- During admission to or selection for postgraduate education or specialization in order to ensure that all populations from the society are represented in the HPE student population. (Mulder et al. 2023)

In HPE research the definition of inclusion would be important in the following areas:

- For ensuring that the HPE literature includes perspectives, learnings and authors from all over the world (Global North as well as Global South). (Naidu 2021)
- For ensuring that Editorial Boards of international HPE journals include members from all over the world (Global North as well as Global South). (Kusurkar 2022)
- For ensuring fair and inclusive review and feedback processes for authors in HPE. (Kusurkar, Lilley & Harden 2024)

**References:**

*Mulder L, Wouters A, Akwiwu EU, Koster AS, Ravesloot JH, Peerdeman SM, Salih M, Croiset G, Kusurkar RA. Diversity in the pathway from medical student to specialist in the Netherlands: a retrospective cohort study. Lancet Reg Heal – Eur 2023; 35:100749.* *https://doi.org/10.1016/j.lanepe.2023.100749* *Isik U, Wouters A, Verdonk P, Croiset G, Kusurkar RA. ‘As an ethnic minority, you just have to work twice as hard.’ Experiences and motivation of ethnic minority students in medical education. Persp Med Educ 2021; 10: 272–278.* *https://link.springer.com/article/10.1007/s40037-021-00679-4* *Muntinga ME, Krajenbrink VQE, Peerdeman SM, Croiset G, Verdonk P. Toward diversity-responsive medical education: taking an intersectionality-based approach to a curriculum evaluation. Adv in Health Sci Educ 2016; 21: 541–559.* *https://doi.org/10.1007/s10459-015-9650-9* *Naidu T. Says who? Northern ventriloquism, or epistemic disobedience in global health scholarship. Lancet Glob Heal 2021; 9: E1332–E5.* *Kusurkar RA. The leaky pipeline of publications and knowledge generation in medical education. Persp Med Educ 2022; 11: 70–72.* *https://doi.org/10.1007/s40037-022-00700-4* *Kusurkar RA, Lilley PM, Harden RM. Medical Teacher’s Equity Diversity Inclusion Policy. Med Teach 2024. Early Online.* *http://dx.doi.org/10.1080/0142159X.2024.2334394*

# Reviewer report: Reviewer response for version 1

**DOI:** 10.21956/mep.21580.r36255

**Published:** 2024-04-12

**Authors:** Diantha Soemantri

**Affiliations:** 1 Universitas Indonesia, Jakarta, Indonesia

This opinion article highlights what it means by term 'inclusion' in the context of medical and health professions education (HPE). The author started by highlighting the literature gap related to inclusion in HPE, especially in regards to the varied definition of inclusion. The author continued by describing what inclusion is based on existing literature and arguing for a more comprehensive meaning of inclusion, in which it is not just related to a sense of belonging but more broadly, it highly correlates with opportunities to participate and contribute in a meaningful way.I think this is a strong and important opinion article, given the issues of Diversity, Equity and Inclusion (DEI), which have been at the forefront in many fields including medical education. Many studies in medical education are now conducted around the issues of DEI hence similar perceptions and understanding regarding what inclusion is helps set the adequate foundation for further discussion. The author has systematically and seamlessly argue for this 'new' definition by drawing on current literature and existing framework related to inclusion.As a minor point to further strengthen the article, I would suggest that the author could add a bit more medical and HPE context in the discussion related to the term 'inclusion'. Although the first paragraph of the article revolves around the medical and HPE context, I feel that the context is a bit lacking at the later part of the article. Perhaps the author could discuss the implications more specifically in medical and HPE context, for example related to selection, assessment, etc.

Is the topic of the opinion article discussed accurately in the context of the current literature?

Yes

Are arguments sufficiently supported by evidence from the published literature?

Yes

Are all factual statements correct and adequately supported by citations?

Yes

Are the conclusions drawn balanced and justified on the basis of the presented arguments?

Yes

Reviewer Expertise:

Assessment, selection and interprofessional education.

I confirm that I have read this submission and believe that I have an appropriate level of expertise to confirm that it is of an acceptable scientific standard, however I have significant reservations, as outlined above.
